# [(*Z*)-*O*-Ethyl *N*-(4-nitro­phen­yl)thio­carbamato-κ*S*](triethyl­phosphine-κ*P*)gold(I)

**DOI:** 10.1107/S1600536809043499

**Published:** 2009-10-28

**Authors:** Soo Yei Ho, Edward R. T. Tiekink

**Affiliations:** a Department of Chemistry, National University of Singapore, Singapore 117543; b Department of Chemistry, University of Malaya, 50603 Kuala Lumpur, Malaysia

## Abstract

In the title compound, [Au(C_9_H_9_N_2_O_3_S)(C_6_H_15_P)], two virtually identical mol­ecules comprise the asymmetric unit. These are connected by Au⋯Au [3.6796 (4) Å] and Au⋯S [3.6325 (18) and 3.5471 (18) Å] contacts, forming a dimeric aggregate. The presence of intra­molecular Au⋯O contacts [2.993 (5) and 2.957 (5) Å] is responsible for the slight deviations from the ideal linear coordination environments about the Au^I^ ions. The conformation about the central C=N double bond is *Z*. Supra­molecular chains sustained by π–π [3.573 (4) Å] and C—H⋯π inter­actions are evident in the crystal structure. These are connected into layers *via* weak inter­molecular C—H⋯O inter­actions involving the nitro-group O atoms.

## Related literature

For structural systematics and luminescence properties of phosphinegold(I) carbonimidothio­ates, see: Ho *et al.* (2006 ▶); Ho & Tiekink (2007 ▶); Kuan *et al.* (2008 ▶). For the synthesis, see: Hall *et al.* (1993 ▶). For the structure analysis, see: Spek (2009 ▶).
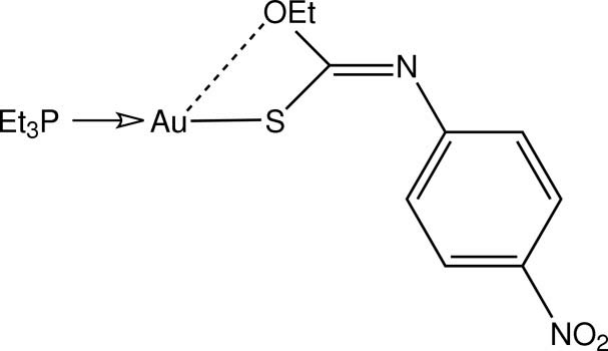


## Experimental

### 

#### Crystal data

[Au(C_9_H_9_N_2_O_3_S)(C_6_H_15_P)]
*M* *_r_* = 540.36
Triclinic, 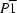

*a* = 11.5340 (6) Å
*b* = 13.7656 (7) Å
*c* = 14.5177 (8) Å
α = 114.223 (2)°
β = 109.374 (2)°
γ = 95.197 (2)°
*V* = 1912.95 (17) Å^3^
*Z* = 4
Mo *K*α radiation
μ = 7.90 mm^−1^
*T* = 223 K
0.16 × 0.13 × 0.05 mm

#### Data collection

Bruker SMART CCD diffractometer
Absorption correction: multi-scan (*SADABS*, Bruker, 2000 ▶) *T*_min_ = 0.584, *T*_max_ = 1
13599 measured reflections
8710 independent reflections
6224 reflections with *I* > 2σ(*I*)
*R*_int_ = 0.033

#### Refinement

*R*[*F* ^2^ > 2σ(*F* ^2^)] = 0.040
*wR*(*F* ^2^) = 0.086
*S* = 0.95
8710 reflections
415 parameters
H-atom parameters constrained
Δρ_max_ = 1.64 e Å^−3^
Δρ_min_ = −0.90 e Å^−3^

### 

Data collection: *SMART* (Bruker, 2000 ▶); cell refinement: *SAINT* (Bruker, 2000 ▶); data reduction: *SAINT*; program(s) used to solve structure: *PATTY* in *DIRDIF92* (Beurskens *et al.*, 1992 ▶); program(s) used to refine structure: *SHELXL97* (Sheldrick, 2008 ▶); molecular graphics: *DIAMOND* (Brandenburg, 2006 ▶); software used to prepare material for publication: *SHELXL97*.

## Supplementary Material

Crystal structure: contains datablocks global, I. DOI: 10.1107/S1600536809043499/lh2932sup1.cif

Structure factors: contains datablocks I. DOI: 10.1107/S1600536809043499/lh2932Isup2.hkl

Additional supplementary materials:  crystallographic information; 3D view; checkCIF report

## Figures and Tables

**Table:** 

Au1—P1	2.2590 (16)
Au1—S1	2.3151 (16)
Au2—P1*A*	2.2596 (16)
Au2—S1*A*	2.3150 (16)

**Table:** 

P1—Au1—S1	176.10 (6)
P1*A*—Au2—S1*A*	174.04 (6)

**Table 2:** Hydrogen-bond geometry (Å, °)

*D*—H⋯*A*	*D*—H	H⋯*A*	*D*⋯*A*	*D*—H⋯*A*
C10—H10a⋯*Cg*	0.98	2.90	3.585 (7)	128
C11a—H11d⋯O2a^i^	0.97	2.41	3.266 (10)	146
C13—H13c⋯O2^ii^	0.97	2.44	3.177 (12)	132
C13a—H13f⋯O3a^iii^	0.97	2.52	3.251 (11)	132
C14—H14b⋯O2^ii^	0.98	2.52	3.444 (9)	157

Symmetry codes: (i) 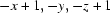; (ii) 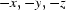; (iii) 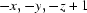. *Cg* is the centroid of the C2*A*–C7*A* ring.

**Fig. 1.**
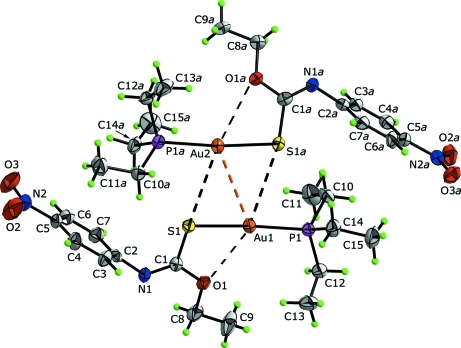
Molecular structures of the two independent molecules comprising the asymmetric unit of (I), showing atom-labelling scheme and displacement ellipsoids at the 50% probability level. The Au···Au and Au···S interactions are shown as orange and black dashed lines, respectively.

**Fig. 2.**
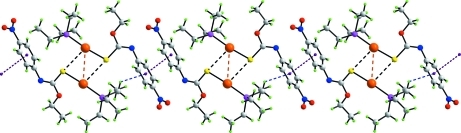
Supramolecular chain formation in (I) mediated by π–π and C—H···π contacts (purple and green dashed lines, respectively). The Au···Au and Au···S interactions are shown as orange and black dashed lines, respectively. Colour code: Au, orange; S, yellow; P, pink; O, red; N, blue; C, grey; and H, green.

**Fig. 3.**
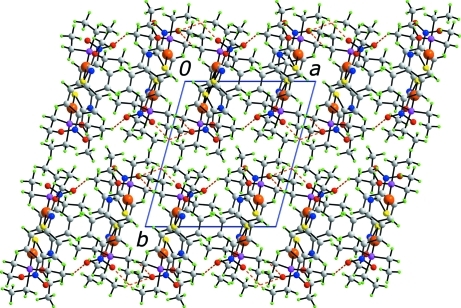
Unit-cell contents for (I) viewed in projection down the c axis. The supramolecular chains illustrated in Fig. 2 are linked by C—H···O interactions (orange dashed lines) to form layers that stack along the b axis. Colour code as for Fig. 2.

## supplementary crystallographic information

### Comment

As part of an on-going study of the structural systematics, including luminescence properties, of phosphinegold(I) carbonimidothioates (Ho *et al.* 2006; Ho & Tiekink, 2007; Kuan *et al.*, 2008), the title compound, (I), was investigated. Two independent molecules comprise the asymmetric unit, Fig. 1, and these are virtually identical as seen in the r.m.s. values: 0.0105 Å for distances and 1.076 ° for angles (Spek, 2009). The molecules are connected by Au1···Au2 interactions, 3.6796 (4) Å, as well as Au1···S1A and Au2···S1 contacts of 3.6325 (18) and 3.5471 (18) Å, respectively, Fig. 1. In accord with expectation, the Au—S bond distances (Au—S = 2.3151 (16) and 2.3150 (16) Å) are longer than the Au—P distances (Au—P = 2.2590 (16) and 2.2596 (16) Å). Deviations from the ideal linear geometry defined by the S and P donor atoms (S—Au—P = 176.10 (6) and 174.04 (6) °) are traced to the close approach of the O1/O1a atoms (2.993 (5) and 2.957 (5) Å). The conformation about the central C1-N1 bond is *Z*. Finally, the C1—S1 (1.745 (7) and 1.769 (7) Å) and C1-N1 (1.276 (7) and 1.272 (8) Å) bond distances indicate that the ligand is binding as a thiolate.

The structure of (I) is isomorphous with the methoxy analogue (Ho *et al.*, 2006) and it is noted that there are no significant differences between comparable geometric parameters around the Au atoms.

Supramolecular chains aligned along the c direction are sustained by π–π [*Cg*1···*Cg*2 = 3.573 (4) Å and the dihedral angle between the rings is 3.7 (3) °, where *Cg*1 and *Cg*2 are the centroids of the C2—C7 and C2a—C7a rings, respectively; i: *x*, *y*, 1 + *z*] and C—H···π interactions, Table 1 and Fig. 2. Chains are linked into layers in the *ac* plane *via* C—H···O interactions, Table 1, where the O atoms are derived from the nitro groups; the O2 atom is bifurcated. Layers stack along the b direction, Fig. 3.

### Experimental

Compound (I) was prepared following the standard literature procedure from the reaction of Et_3_PAuCl and EtOC(S)N(H)C_6_H_4_NO_2_-4 in the presence of base (Hall *et al.*, 1993). Yellow crystals were obtained from the layering of ethanol on a dichloromethane solution of (I); m. pt. 378–379 K. Analysis for C_15_H_24_AuN_2_O_3_PS: found (calculated): C: 33.57 (33.34); H: 4.80 (4.48); N: 5.09 (5.18); S: 5.61 (5.93). IR (cm^-1^): ν(C—S) 1102 s, 849*m*; ν(C—N) 1574*m*; ν(C—O) 1152 s. ^31^P{^1^H} NMR: δ 36.4 p.p.m.

### Refinement

The H atoms were geometrically placed (C—H = 0.94–0.98 Å) and refined as riding with *U*_iso_(H) = 1.2–1.5*U*_eq_(C). The maximum and minimum residual electron density peaks of 1.64 and 0.90 e Å^-3^, respectively, were located 0.93 Å and 1.47 Å from the Au2 and Au1 atoms, respectively.

### Crystal data

**Table:**

[Au(C_9_H_9_N_2_O_3_S)(C_6_H_15_P)]	*Z* = 4
*M**_r_* = 540.36	*F*(000) = 1048
Triclinic, *P*1	*D*_x_ = 1.876 Mg m^−^^3^
Hall symbol: -P 1	Mo *K*α radiation, λ = 0.71069 Å
*a* = 11.5340 (6) Å	Cell parameters from 2986 reflections
*b* = 13.7656 (7) Å	θ = 2.4–25.2°
*c* = 14.5177 (8) Å	µ = 7.90 mm^−^^1^
α = 114.223 (2)°	*T* = 223 K
β = 109.374 (2)°	Block, pale-yellow
γ = 95.197 (2)°	0.16 × 0.13 × 0.05 mm
*V* = 1912.95 (17) Å^3^	

### Data collection

**Table:**

Bruker SMART CCD diffractometer	8710 independent reflections
Radiation source: fine-focus sealed tube	6224 reflections with *I* > 2σ(*I*)
graphite	*R*_int_ = 0.033
ω' scans	θ_max_ = 27.5°, θ_min_ = 1.7°
Absorption correction: multi-scan (*SADABS*, Bruker, 2000)	*h* = −14→14
*T*_min_ = 0.584, *T*_max_ = 1	*k* = −17→17
13599 measured reflections	*l* = −17→18

### Refinement

**Table:**

Refinement on *F*^2^	Primary atom site location: structure-invariant direct methods
Least-squares matrix: full	Secondary atom site location: difference Fourier map
*R*[*F*^2^ > 2σ(*F*^2^)] = 0.040	Hydrogen site location: inferred from neighbouring sites
*wR*(*F*^2^) = 0.086	H-atom parameters constrained
*S* = 0.95	*w* = 1/[σ^2^(*F*_o_^2^) + (0.0239*P*)^2^] where *P* = (*F*_o_^2^ + 2*F*_c_^2^)/3
8710 reflections	(Δ/σ)_max_ = 0.001
415 parameters	Δρ_max_ = 1.64 e Å^−^^3^
0 restraints	Δρ_min_ = −0.90 e Å^−^^3^

### Special details

**Table:**

Geometry. All s.u.'s (except the s.u. in the dihedral angle between two l.s. planes) are estimated using the full covariance matrix. The cell s.u.'s are taken into account individually in the estimation of s.u.'s in distances, angles and torsion angles; correlations between s.u.'s in cell parameters are only used when they are defined by crystal symmetry. An approximate (isotropic) treatment of cell s.u.'s is used for estimating s.u.'s involving l.s. planes.
Refinement. Refinement of *F*^2^ against ALL reflections. The weighted *R*-factor *wR* and goodness of fit *S* are based on *F*^2^, conventional *R*-factors *R* are based on *F*, with *F* set to zero for negative *F*^2^. The threshold expression of *F*^2^ > 2σ(*F*^2^) is used only for calculating *R*-factors(gt) *etc*. and is not relevant to the choice of reflections for refinement. *R*-factors based on *F*^2^ are statistically about twice as large as those based on *F*, and *R*- factors based on ALL data will be even larger.

### Fractional atomic coordinates and isotropic or equivalent isotropic displacement parameters (Å^2^)

**Table:**

	*x*	*y*	*z*	*U*_iso_*/*U*_eq_	
Au1	0.21328 (2)	−0.09791 (2)	0.25089 (2)	0.03076 (8)	
Au2	0.19099 (2)	0.18507 (2)	0.39905 (2)	0.03003 (8)	
S1	0.22021 (16)	0.00121 (13)	0.15604 (14)	0.0331 (4)	
S1A	0.18420 (16)	0.09210 (14)	0.49949 (14)	0.0351 (4)	
P1	0.20592 (16)	−0.20492 (14)	0.33338 (14)	0.0307 (4)	
P1A	0.20508 (16)	0.29223 (14)	0.31779 (14)	0.0305 (4)	
O1	0.2526 (4)	−0.1897 (3)	0.0409 (4)	0.0329 (10)	
O1A	0.2993 (5)	0.2989 (4)	0.6434 (4)	0.0411 (12)	
O2	0.1567 (5)	0.3470 (5)	−0.0866 (5)	0.0705 (18)	
O3A	0.1677 (5)	−0.2800 (4)	0.6929 (4)	0.0549 (15)	
O3	0.3592 (6)	0.4014 (5)	0.0034 (5)	0.0708 (19)	
O2A	0.3722 (5)	−0.2475 (4)	0.7657 (4)	0.0536 (14)	
N1	0.2607 (5)	−0.0715 (4)	−0.0314 (4)	0.0326 (13)	
N1A	0.3305 (5)	0.1841 (4)	0.7206 (4)	0.0371 (14)	
N2	0.2585 (6)	0.3328 (5)	−0.0391 (5)	0.0425 (15)	
N2A	0.2761 (6)	−0.2217 (5)	0.7277 (4)	0.0374 (14)	
C1	0.2458 (6)	−0.0905 (5)	0.0435 (5)	0.0282 (14)	
C1A	0.2794 (6)	0.1971 (5)	0.6356 (6)	0.0374 (16)	
C2	0.2575 (6)	0.0314 (5)	−0.0288 (5)	0.0306 (15)	
C2A	0.3127 (6)	0.0805 (5)	0.7168 (5)	0.0331 (15)	
C3	0.1428 (6)	0.0564 (6)	−0.0681 (6)	0.0362 (16)	
H3	0.0653	0.0060	−0.0922	0.043*	
C3A	0.4203 (6)	0.0442 (6)	0.7526 (5)	0.0339 (15)	
H3A	0.5018	0.0878	0.7750	0.041*	
C4	0.1422 (6)	0.1551 (6)	−0.0718 (6)	0.0382 (17)	
H4	0.0649	0.1720	−0.0988	0.046*	
C4A	0.4078 (6)	−0.0544 (5)	0.7553 (5)	0.0339 (15)	
H4A	0.4804	−0.0785	0.7791	0.041*	
C5	0.2567 (6)	0.2285 (5)	−0.0353 (5)	0.0302 (14)	
C5A	0.2892 (6)	−0.1177 (5)	0.7234 (5)	0.0310 (15)	
C6	0.3712 (6)	0.2065 (6)	0.0051 (5)	0.0377 (16)	
H6	0.4483	0.2580	0.0304	0.045*	
C6A	0.1817 (6)	−0.0840 (6)	0.6884 (5)	0.0357 (16)	
H6A	0.1011	−0.1284	0.6667	0.043*	
C7	0.3713 (6)	0.1074 (6)	0.0080 (6)	0.0363 (16)	
H7	0.4491	0.0912	0.0351	0.044*	
C7A	0.1920 (6)	0.0144 (6)	0.6850 (5)	0.0351 (16)	
H7A	0.1186	0.0377	0.6615	0.042*	
C8	0.2844 (7)	−0.2662 (6)	−0.0443 (6)	0.0442 (18)	
H8A	0.3585	−0.2291	−0.0474	0.053*	
H8B	0.2126	−0.2968	−0.1165	0.053*	
C8A	0.3857 (8)	0.3883 (6)	0.7495 (6)	0.054 (2)	
H8A1	0.4665	0.3704	0.7763	0.065*	
H8A2	0.3485	0.4027	0.8040	0.065*	
C9	0.3139 (10)	−0.3567 (7)	−0.0141 (8)	0.077 (3)	
H9A	0.3352	−0.4111	−0.0697	0.115*	
H9B	0.2401	−0.3920	−0.0104	0.115*	
H9C	0.3855	−0.3253	0.0572	0.115*	
C9A	0.4063 (10)	0.4857 (6)	0.7313 (7)	0.081 (3)	
H9A1	0.4642	0.5487	0.8004	0.121*	
H9A2	0.3255	0.5022	0.7047	0.121*	
H9A3	0.4427	0.4699	0.6770	0.121*	
C10	0.3378 (6)	−0.1555 (6)	0.4659 (5)	0.0400 (17)	
H10A	0.3351	−0.0828	0.5166	0.048*	
H10B	0.3266	−0.2058	0.4962	0.048*	
C10A	0.3475 (6)	0.3029 (6)	0.2904 (6)	0.0400 (17)	
H10C	0.3378	0.2343	0.2263	0.048*	
H10D	0.3562	0.3633	0.2717	0.048*	
C11	0.4678 (7)	−0.1459 (7)	0.4621 (7)	0.058 (2)	
H11A	0.5330	−0.1194	0.5358	0.087*	
H11B	0.4812	−0.0945	0.4344	0.087*	
H11C	0.4724	−0.2178	0.4135	0.087*	
C11A	0.4676 (7)	0.3239 (8)	0.3881 (7)	0.060 (2)	
H11D	0.5405	0.3287	0.3695	0.091*	
H11E	0.4602	0.2637	0.4061	0.091*	
H11F	0.4788	0.3927	0.4513	0.091*	
C12	0.2184 (7)	−0.3419 (6)	0.2494 (6)	0.0431 (18)	
H12A	0.2997	−0.3343	0.2419	0.052*	
H12B	0.2188	−0.3862	0.2878	0.052*	
C12A	0.2216 (6)	0.4347 (5)	0.4128 (6)	0.0366 (16)	
H12C	0.3018	0.4611	0.4780	0.044*	
H12D	0.2268	0.4805	0.3767	0.044*	
C13	0.1121 (8)	−0.4019 (6)	0.1364 (6)	0.057 (2)	
H13A	0.1245	−0.4732	0.0955	0.086*	
H13B	0.1115	−0.3589	0.0975	0.086*	
H13C	0.0315	−0.4123	0.1430	0.086*	
C13A	0.1150 (7)	0.4504 (6)	0.4499 (7)	0.054 (2)	
H13D	0.1305	0.5278	0.5002	0.081*	
H13E	0.1100	0.4068	0.4874	0.081*	
H13F	0.0353	0.4268	0.3862	0.081*	
C14	0.0625 (6)	−0.2223 (6)	0.3572 (6)	0.0383 (17)	
H14A	0.0647	−0.1512	0.4143	0.046*	
H14B	−0.0107	−0.2402	0.2891	0.046*	
C14A	0.0717 (6)	0.2537 (6)	0.1887 (5)	0.0370 (16)	
H14C	0.0734	0.1843	0.1320	0.044*	
H14D	−0.0074	0.2400	0.1981	0.044*	
C15	0.0394 (8)	−0.3102 (7)	0.3926 (7)	0.061 (2)	
H15A	−0.0397	−0.3122	0.4025	0.091*	
H15B	0.1093	−0.2923	0.4616	0.091*	
H15C	0.0341	−0.3818	0.3360	0.091*	
C15A	0.0686 (7)	0.3393 (7)	0.1463 (6)	0.054 (2)	
H15D	−0.0056	0.3119	0.0773	0.080*	
H15E	0.1452	0.3521	0.1343	0.080*	
H15F	0.0642	0.4080	0.2006	0.080*	

### Atomic displacement parameters (Å^2^)

**Table:**

	*U*^11^	*U*^22^	*U*^33^	*U*^12^	*U*^13^	*U*^23^
Au1	0.03699 (15)	0.03127 (15)	0.02879 (15)	0.00955 (12)	0.01461 (12)	0.01725 (13)
Au2	0.03449 (14)	0.02922 (15)	0.02933 (15)	0.00682 (12)	0.01326 (12)	0.01643 (12)
S1	0.0474 (10)	0.0267 (9)	0.0347 (9)	0.0131 (8)	0.0225 (8)	0.0176 (8)
S1A	0.0467 (10)	0.0258 (9)	0.0294 (9)	0.0025 (8)	0.0103 (8)	0.0154 (8)
P1	0.0411 (9)	0.0286 (9)	0.0275 (9)	0.0132 (8)	0.0156 (8)	0.0155 (8)
P1A	0.0381 (9)	0.0249 (9)	0.0307 (9)	0.0055 (8)	0.0158 (8)	0.0142 (8)
O1	0.047 (3)	0.023 (2)	0.037 (3)	0.016 (2)	0.023 (2)	0.016 (2)
O1A	0.062 (3)	0.023 (2)	0.029 (3)	0.005 (2)	0.012 (2)	0.011 (2)
O2	0.068 (4)	0.049 (4)	0.082 (4)	0.022 (3)	0.002 (3)	0.040 (4)
O3A	0.055 (3)	0.039 (3)	0.054 (3)	−0.006 (3)	0.008 (3)	0.022 (3)
O3	0.067 (4)	0.040 (3)	0.088 (5)	0.003 (3)	0.005 (4)	0.038 (4)
O2A	0.066 (3)	0.055 (3)	0.062 (4)	0.028 (3)	0.030 (3)	0.041 (3)
N1	0.045 (3)	0.028 (3)	0.035 (3)	0.010 (3)	0.020 (3)	0.020 (3)
N1A	0.055 (4)	0.024 (3)	0.031 (3)	0.010 (3)	0.014 (3)	0.015 (3)
N2	0.057 (4)	0.032 (3)	0.039 (4)	0.015 (3)	0.014 (3)	0.021 (3)
N2A	0.049 (4)	0.034 (3)	0.029 (3)	0.007 (3)	0.013 (3)	0.017 (3)
C1	0.031 (3)	0.027 (3)	0.029 (3)	0.008 (3)	0.012 (3)	0.015 (3)
C1A	0.048 (4)	0.026 (4)	0.034 (4)	0.006 (3)	0.017 (3)	0.011 (3)
C2	0.048 (4)	0.031 (4)	0.022 (3)	0.018 (3)	0.021 (3)	0.014 (3)
C2A	0.048 (4)	0.026 (3)	0.021 (3)	0.005 (3)	0.014 (3)	0.008 (3)
C3	0.035 (4)	0.033 (4)	0.045 (4)	0.006 (3)	0.019 (3)	0.021 (3)
C3A	0.037 (4)	0.036 (4)	0.027 (3)	0.002 (3)	0.009 (3)	0.016 (3)
C4	0.037 (4)	0.041 (4)	0.042 (4)	0.015 (3)	0.013 (3)	0.026 (4)
C4A	0.037 (4)	0.038 (4)	0.027 (3)	0.014 (3)	0.013 (3)	0.015 (3)
C5	0.042 (4)	0.026 (3)	0.028 (3)	0.011 (3)	0.014 (3)	0.017 (3)
C5A	0.040 (4)	0.025 (3)	0.028 (3)	0.007 (3)	0.014 (3)	0.014 (3)
C6	0.038 (4)	0.034 (4)	0.036 (4)	0.006 (3)	0.011 (3)	0.015 (3)
C6A	0.033 (3)	0.043 (4)	0.036 (4)	0.001 (3)	0.017 (3)	0.022 (3)
C7	0.032 (3)	0.035 (4)	0.038 (4)	0.006 (3)	0.012 (3)	0.017 (3)
C7A	0.038 (4)	0.039 (4)	0.030 (4)	0.013 (3)	0.013 (3)	0.016 (3)
C8	0.062 (5)	0.031 (4)	0.055 (5)	0.024 (4)	0.038 (4)	0.021 (4)
C8A	0.083 (6)	0.033 (4)	0.032 (4)	0.001 (4)	0.014 (4)	0.013 (4)
C9	0.132 (8)	0.046 (5)	0.117 (8)	0.054 (6)	0.097 (7)	0.054 (6)
C9A	0.138 (9)	0.028 (4)	0.037 (5)	−0.018 (5)	0.011 (5)	0.007 (4)
C10	0.046 (4)	0.049 (5)	0.026 (4)	0.018 (4)	0.013 (3)	0.020 (3)
C10A	0.051 (4)	0.033 (4)	0.052 (5)	0.010 (4)	0.033 (4)	0.024 (4)
C11	0.043 (4)	0.076 (6)	0.056 (5)	0.017 (4)	0.012 (4)	0.037 (5)
C11A	0.039 (4)	0.079 (6)	0.061 (6)	0.013 (4)	0.023 (4)	0.030 (5)
C12	0.063 (5)	0.033 (4)	0.035 (4)	0.015 (4)	0.021 (4)	0.016 (3)
C12A	0.043 (4)	0.024 (3)	0.039 (4)	0.006 (3)	0.022 (3)	0.008 (3)
C13	0.083 (6)	0.039 (5)	0.041 (5)	0.022 (5)	0.026 (5)	0.010 (4)
C13A	0.066 (5)	0.031 (4)	0.068 (6)	0.018 (4)	0.038 (5)	0.017 (4)
C14	0.032 (3)	0.044 (4)	0.042 (4)	0.007 (3)	0.015 (3)	0.023 (4)
C14A	0.039 (4)	0.036 (4)	0.034 (4)	0.003 (3)	0.011 (3)	0.019 (3)
C15	0.073 (6)	0.056 (5)	0.073 (6)	0.011 (5)	0.039 (5)	0.042 (5)
C15A	0.057 (5)	0.061 (5)	0.048 (5)	0.015 (4)	0.015 (4)	0.035 (4)

### Geometric parameters (Å, °)

**Table:**

Au1—P1	2.2590 (16)	C8—H8A	0.9800
Au1—S1	2.3151 (16)	C8—H8B	0.9800
Au2—P1A	2.2596 (16)	C8A—C9A	1.482 (10)
Au2—S1A	2.3150 (16)	C8A—H8A1	0.9800
S1—C1	1.745 (7)	C8A—H8A2	0.9800
S1A—C1A	1.769 (7)	C9—H9A	0.9700
P1—C10	1.810 (6)	C9—H9B	0.9700
P1—C14	1.814 (6)	C9—H9C	0.9700
P1—C12	1.828 (7)	C9A—H9A1	0.9700
P1A—C14A	1.813 (6)	C9A—H9A2	0.9700
P1A—C10A	1.821 (7)	C9A—H9A3	0.9700
P1A—C12A	1.823 (7)	C10—C11	1.515 (10)
O1—C1	1.361 (7)	C10—H10A	0.9800
O1—C8	1.441 (8)	C10—H10B	0.9800
O1A—C1A	1.350 (7)	C10A—C11A	1.518 (10)
O1A—C8A	1.444 (8)	C10A—H10C	0.9800
O2—N2	1.228 (7)	C10A—H10D	0.9800
O3A—N2A	1.237 (7)	C11—H11A	0.9700
O3—N2	1.212 (7)	C11—H11B	0.9700
O2A—N2A	1.219 (7)	C11—H11C	0.9700
N1—C1	1.276 (7)	C11A—H11D	0.9700
N1—C2	1.406 (8)	C11A—H11E	0.9700
N1A—C1A	1.272 (8)	C11A—H11F	0.9700
N1A—C2A	1.399 (8)	C12—C13	1.499 (9)
N2—C5	1.459 (8)	C12—H12A	0.9800
N2A—C5A	1.455 (8)	C12—H12B	0.9800
C2—C7	1.389 (8)	C12A—C13A	1.497 (9)
C2—C3	1.392 (9)	C12A—H12C	0.9800
C2A—C3A	1.399 (9)	C12A—H12D	0.9800
C2A—C7A	1.406 (9)	C13—H13A	0.9700
C3—C4	1.382 (9)	C13—H13B	0.9700
C3—H3	0.9400	C13—H13C	0.9700
C3A—C4A	1.369 (9)	C13A—H13D	0.9700
C3A—H3A	0.9400	C13A—H13E	0.9700
C4—C5	1.378 (8)	C13A—H13F	0.9700
C4—H4	0.9400	C14—C15	1.529 (9)
C4A—C5A	1.370 (8)	C14—H14A	0.9800
C4A—H4A	0.9400	C14—H14B	0.9800
C5—C6	1.372 (9)	C14A—C15A	1.534 (9)
C5A—C6A	1.375 (9)	C14A—H14C	0.9800
C6—C7	1.382 (9)	C14A—H14D	0.9800
C6—H6	0.9400	C15—H15A	0.9700
C6A—C7A	1.371 (9)	C15—H15B	0.9700
C6A—H6A	0.9400	C15—H15C	0.9700
C7—H7	0.9400	C15A—H15D	0.9700
C7A—H7A	0.9400	C15A—H15E	0.9700
C8—C9	1.513 (10)	C15A—H15F	0.9700
			
P1—Au1—S1	176.10 (6)	H9A—C9—H9B	109.5
P1A—Au2—S1A	174.04 (6)	C8—C9—H9C	109.5
C1—S1—Au1	102.9 (2)	H9A—C9—H9C	109.5
C1A—S1A—Au2	100.9 (2)	H9B—C9—H9C	109.5
C10—P1—C14	106.0 (3)	C8A—C9A—H9A1	109.5
C10—P1—C12	104.1 (3)	C8A—C9A—H9A2	109.5
C14—P1—C12	108.0 (3)	H9A1—C9A—H9A2	109.5
C10—P1—Au1	113.9 (2)	C8A—C9A—H9A3	109.5
C14—P1—Au1	114.2 (2)	H9A1—C9A—H9A3	109.5
C12—P1—Au1	109.9 (2)	H9A2—C9A—H9A3	109.5
C14A—P1A—C10A	106.1 (3)	C11—C10—P1	114.5 (5)
C14A—P1A—C12A	107.7 (3)	C11—C10—H10A	108.6
C10A—P1A—C12A	102.7 (3)	P1—C10—H10A	108.6
C14A—P1A—Au2	115.6 (2)	C11—C10—H10B	108.6
C10A—P1A—Au2	114.5 (2)	P1—C10—H10B	108.6
C12A—P1A—Au2	109.2 (2)	H10A—C10—H10B	107.6
C1—O1—C8	117.1 (5)	C11A—C10A—P1A	112.6 (5)
C1A—O1A—C8A	116.8 (5)	C11A—C10A—H10C	109.1
C1—N1—C2	120.6 (6)	P1A—C10A—H10C	109.1
C1A—N1A—C2A	122.3 (6)	C11A—C10A—H10D	109.1
O3—N2—O2	122.6 (6)	P1A—C10A—H10D	109.1
O3—N2—C5	119.4 (6)	H10C—C10A—H10D	107.8
O2—N2—C5	118.0 (6)	C10—C11—H11A	109.5
O2A—N2A—O3A	122.8 (6)	C10—C11—H11B	109.5
O2A—N2A—C5A	118.7 (6)	H11A—C11—H11B	109.5
O3A—N2A—C5A	118.5 (6)	C10—C11—H11C	109.5
N1—C1—O1	119.6 (6)	H11A—C11—H11C	109.5
N1—C1—S1	126.4 (5)	H11B—C11—H11C	109.5
O1—C1—S1	114.0 (4)	C10A—C11A—H11D	109.5
N1A—C1A—O1A	120.1 (6)	C10A—C11A—H11E	109.5
N1A—C1A—S1A	126.7 (5)	H11D—C11A—H11E	109.5
O1A—C1A—S1A	113.1 (5)	C10A—C11A—H11F	109.5
C7—C2—C3	119.2 (6)	H11D—C11A—H11F	109.5
C7—C2—N1	119.1 (6)	H11E—C11A—H11F	109.5
C3—C2—N1	121.5 (6)	C13—C12—P1	113.3 (5)
N1A—C2A—C3A	118.4 (6)	C13—C12—H12A	108.9
N1A—C2A—C7A	122.7 (6)	P1—C12—H12A	108.9
C3A—C2A—C7A	118.7 (6)	C13—C12—H12B	108.9
C4—C3—C2	120.3 (6)	P1—C12—H12B	108.9
C4—C3—H3	119.8	H12A—C12—H12B	107.7
C2—C3—H3	119.8	C13A—C12A—P1A	114.2 (5)
C4A—C3A—C2A	120.4 (6)	C13A—C12A—H12C	108.7
C4A—C3A—H3A	119.8	P1A—C12A—H12C	108.7
C2A—C3A—H3A	119.8	C13A—C12A—H12D	108.7
C5—C4—C3	119.0 (6)	P1A—C12A—H12D	108.7
C5—C4—H4	120.5	H12C—C12A—H12D	107.6
C3—C4—H4	120.5	C12—C13—H13A	109.5
C3A—C4A—C5A	119.9 (6)	C12—C13—H13B	109.5
C3A—C4A—H4A	120.1	H13A—C13—H13B	109.5
C5A—C4A—H4A	120.1	C12—C13—H13C	109.5
C6—C5—C4	121.9 (6)	H13A—C13—H13C	109.5
C6—C5—N2	118.1 (6)	H13B—C13—H13C	109.5
C4—C5—N2	120.0 (6)	C12A—C13A—H13D	109.5
C4A—C5A—C6A	121.0 (6)	C12A—C13A—H13E	109.5
C4A—C5A—N2A	119.8 (6)	H13D—C13A—H13E	109.5
C6A—C5A—N2A	119.1 (6)	C12A—C13A—H13F	109.5
C5—C6—C7	118.9 (6)	H13D—C13A—H13F	109.5
C5—C6—H6	120.5	H13E—C13A—H13F	109.5
C7—C6—H6	120.5	C15—C14—P1	116.9 (5)
C7A—C6A—C5A	120.1 (6)	C15—C14—H14A	108.1
C7A—C6A—H6A	119.9	P1—C14—H14A	108.1
C5A—C6A—H6A	119.9	C15—C14—H14B	108.1
C6—C7—C2	120.6 (6)	P1—C14—H14B	108.1
C6—C7—H7	119.7	H14A—C14—H14B	107.3
C2—C7—H7	119.7	C15A—C14A—P1A	115.2 (4)
C6A—C7A—C2A	119.8 (6)	C15A—C14A—H14C	108.5
C6A—C7A—H7A	120.1	P1A—C14A—H14C	108.5
C2A—C7A—H7A	120.1	C15A—C14A—H14D	108.5
O1—C8—C9	106.3 (6)	P1A—C14A—H14D	108.5
O1—C8—H8A	110.5	H14C—C14A—H14D	107.5
C9—C8—H8A	110.5	C14—C15—H15A	109.5
O1—C8—H8B	110.5	C14—C15—H15B	109.5
C9—C8—H8B	110.5	H15A—C15—H15B	109.5
H8A—C8—H8B	108.7	C14—C15—H15C	109.5
O1A—C8A—C9A	105.9 (6)	H15A—C15—H15C	109.5
O1A—C8A—H8A1	110.6	H15B—C15—H15C	109.5
C9A—C8A—H8A1	110.6	C14A—C15A—H15D	109.5
O1A—C8A—H8A2	110.6	C14A—C15A—H15E	109.5
C9A—C8A—H8A2	110.6	H15D—C15A—H15E	109.5
H8A1—C8A—H8A2	108.7	C14A—C15A—H15F	109.5
C8—C9—H9A	109.5	H15D—C15A—H15F	109.5
C8—C9—H9B	109.5	H15E—C15A—H15F	109.5

### Hydrogen-bond geometry (Å, °)

**Table:**

*D*—H···*A*	*D*—H	H···*A*	*D*···*A*	*D*—H···*A*
C10—H10a···Cg	0.98	2.90	3.585 (7)	128
C11a—H11d···O2a^i^	0.97	2.41	3.266 (10)	146
C13—H13c···O2^ii^	0.97	2.44	3.177 (12)	132
C13a—H13f···O3a^iii^	0.97	2.52	3.251 (11)	132
C14—H14b···O2^ii^	0.98	2.52	3.444 (9)	157

Symmetry codes:  (i) −*x*+1, −*y*, −*z*+1; (ii) −*x*, −*y*, −*z*; (iii) −*x*, −*y*, −*z*+1.
